# Cannabinoid spoilage, metabolism and cannabidiol(CBD) conversion to Tetrahydrocannabinol(THC) mechanisms with energetic parameters

**DOI:** 10.1186/s42238-024-00239-7

**Published:** 2025-02-10

**Authors:** Alwyn Henriques

**Affiliations:** https://www.linkedin.com/in/alwyn-henriques-270737136?trk=contact-info

**Keywords:** Tetrahydrocannabinol (THC), Cannabidiol (CBD), Cannabinol (CBN), Storage, 11-nor-9-carboxy, Glucuronidation

## Abstract

Chemical redox mechanisms and thermodynamic parameters of the Cannabinoids Δ^9^-Tetrahydrocannabinol (THC) and cannabidiol (CBD) were determined theoretically and using activated complex theory then compared to physical experimentations of chemical spoilage by Pholsiri T et al. and intramolecular conversion of CBD to thc by Daniels. R. et al. Thermodynamic parameters were derived from activated complex theory using standard bond enthalpy data from the elments of Physical Chemistry by Atkins ((Atkins, Elements of Physical Chemistry, 2001)). Situations where psychotropic cannabinoids found in CBD based products were also understood due to the parameters that drive the reduction process of conversion to THC. The metabolism process of cannabinoids has been detailed from consumption to excretion with mechanistic support to deactivation and glucuronidation in order to have a cannabis standard established.

## Introduction

Δ^9^-THC undergoes oxidation which converts to CBN (cannabinol) which is a more stable thermodynamic compound compared to 11-OH THC a more potent cannabinoid. As the product of oxidation at isobaric conditions, cannabidiol follows a similar oxidation pathway to CBND however the cannabinoid undergoes proton catalysis to form THC, a more feasible compound. A pathway is proposed for the decomposition of Δ^9^-tetrahydrocannabinol (I) and its Δ8-isomer (IX) with the eventual formation of cannabinol (II) through epoxy and hydroxylated intermediates (Turner and Elsohly 1979). The goal is to fully understand the metabolism process using thermodynamic parameters to supplement the pharmacokinetics of the enzyme substrate complex cytochrome P450 enzymatic cycle while maintaining charge neutrality during molecular transformation of the cannabinoid during and after the enzyme complex metabolism. The activation energies for the redox of the cannabinoids CBD and Δ^9^- THC were sought after by means of activation energy complex theory to simulate a theoretical value and rate constants for the chemical change. Standard data was then to be compared to experimental energy changes of the cannabinoids CBD and Δ^9^- THC respectively as redox from Polori and Omir respectively. Provided with the energy changes by each mechanism, pharmacokinetics essays and chemical spoilage experimentation could have more understanding in regards to the order of the reactants.

Data provided propose mechanisms and thermodynamic parameters; oxidation of Δ^9^- THC to CBN and CBD to CBND, the model of mammalian metabolism of Δ^9^-THC and CBD in stomach acid and deactivation to 11-nor-carboxy-THC and 7-OH-CBD as primary metabolites, lastly the conversion of CBD to Δ^9^-THC consuming water in an acidic condition.

## Method

Applying bond enthalpy mechanistic change data from Elements of Physical Chemistry by Peter Atkins (Figs. 1, 2, 3, 4, 5, 6, 7 and 8).

**Fig. 1:**
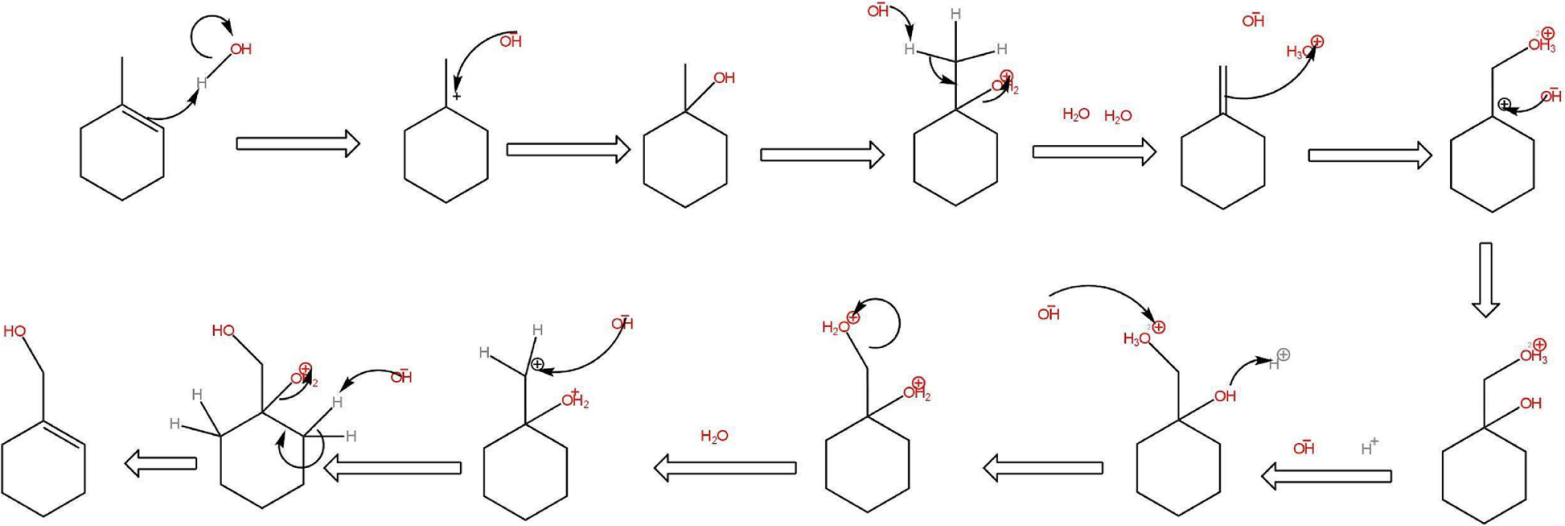
Mechanism of Δ^9^-Tetrahydrocannabinol (THC) reduction to 11-OH-THC

**Fig. 2:**
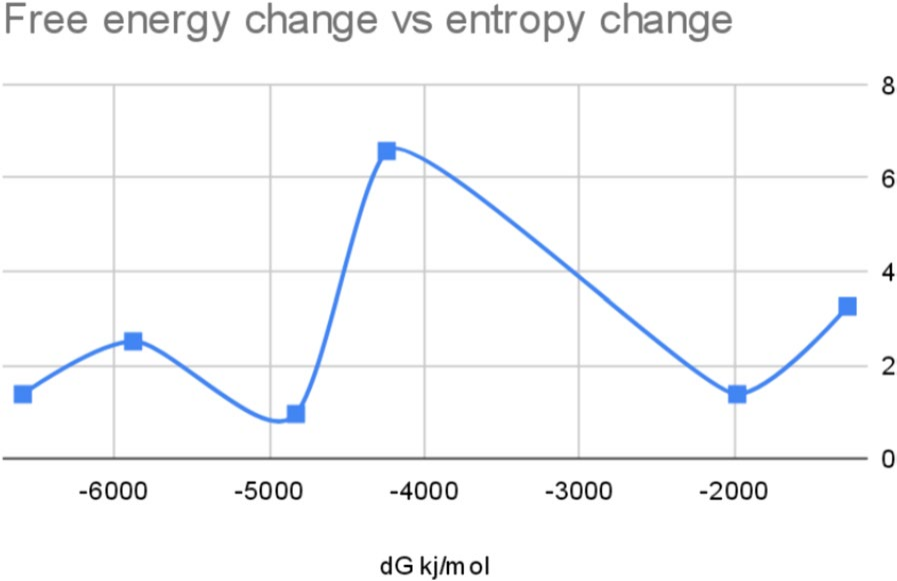
Energy diagram of formation of 11-OH-THC

**Fig. 3:**
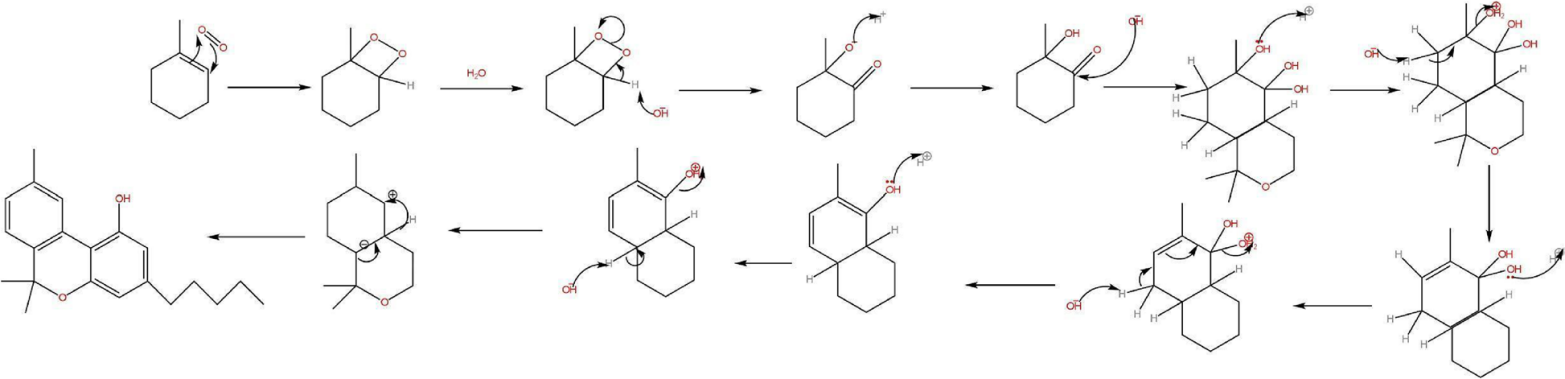
Δ^9^-Tetrahydrocannabinol (Δ.^9^-THC) oxidation mechanism to cannabinol (CBN)

**Fig. 4:**
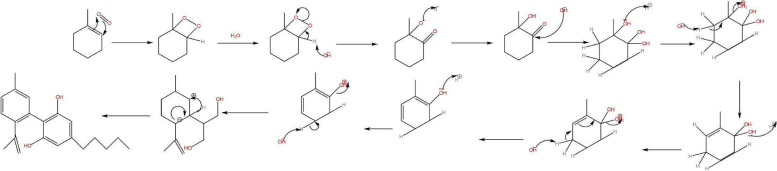
CBD oxidation to CBND mechanism

**Fig. 5:**
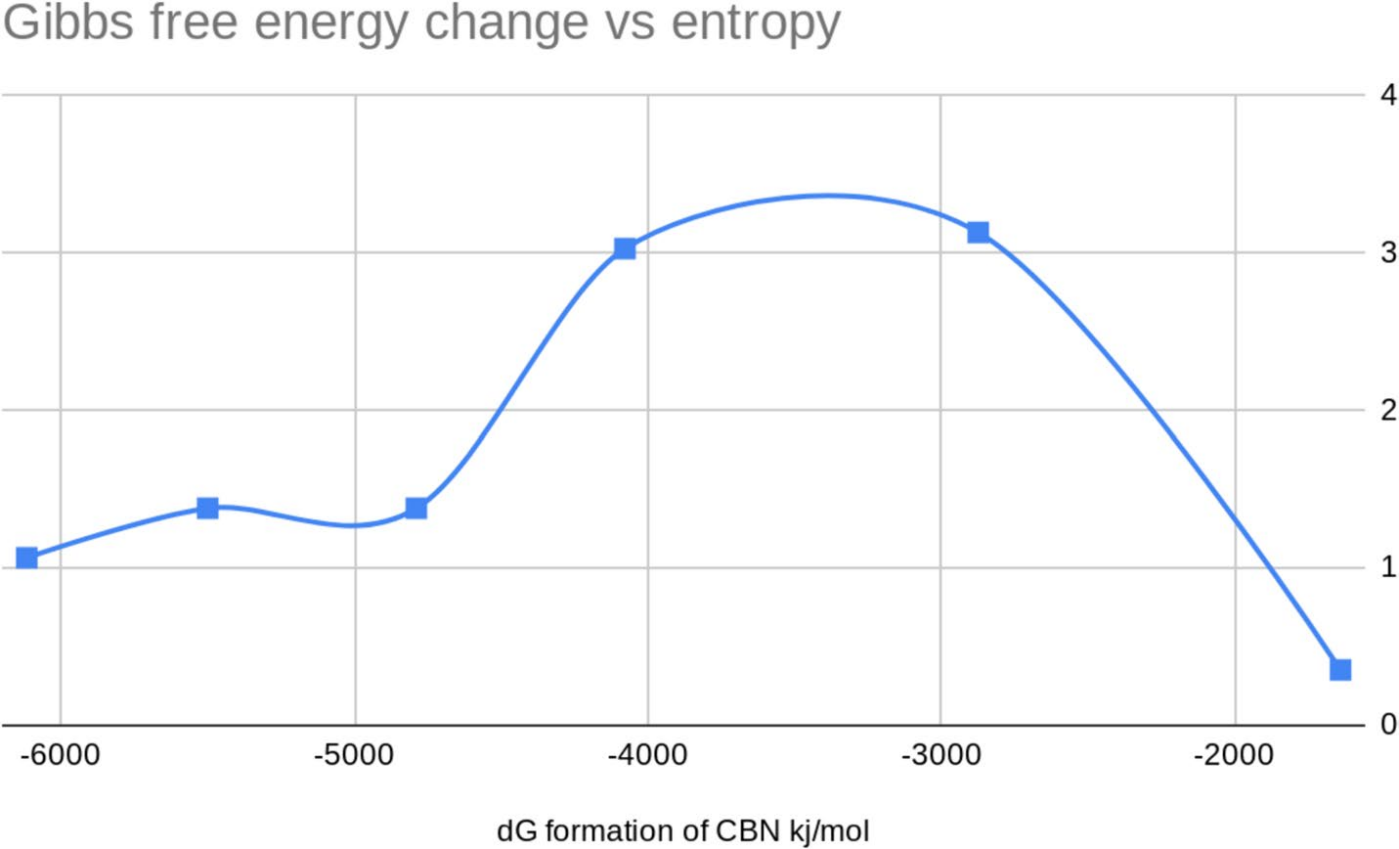
Energy diagram of formation of CBN

**Fig. 6:**
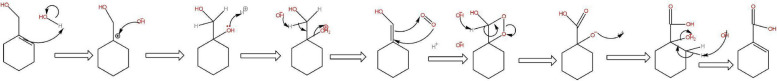
Formation of 11-nor-carboxy-THC from Δ^9^-Tetrahydrocannabinol mechanism

**Fig. 7:**
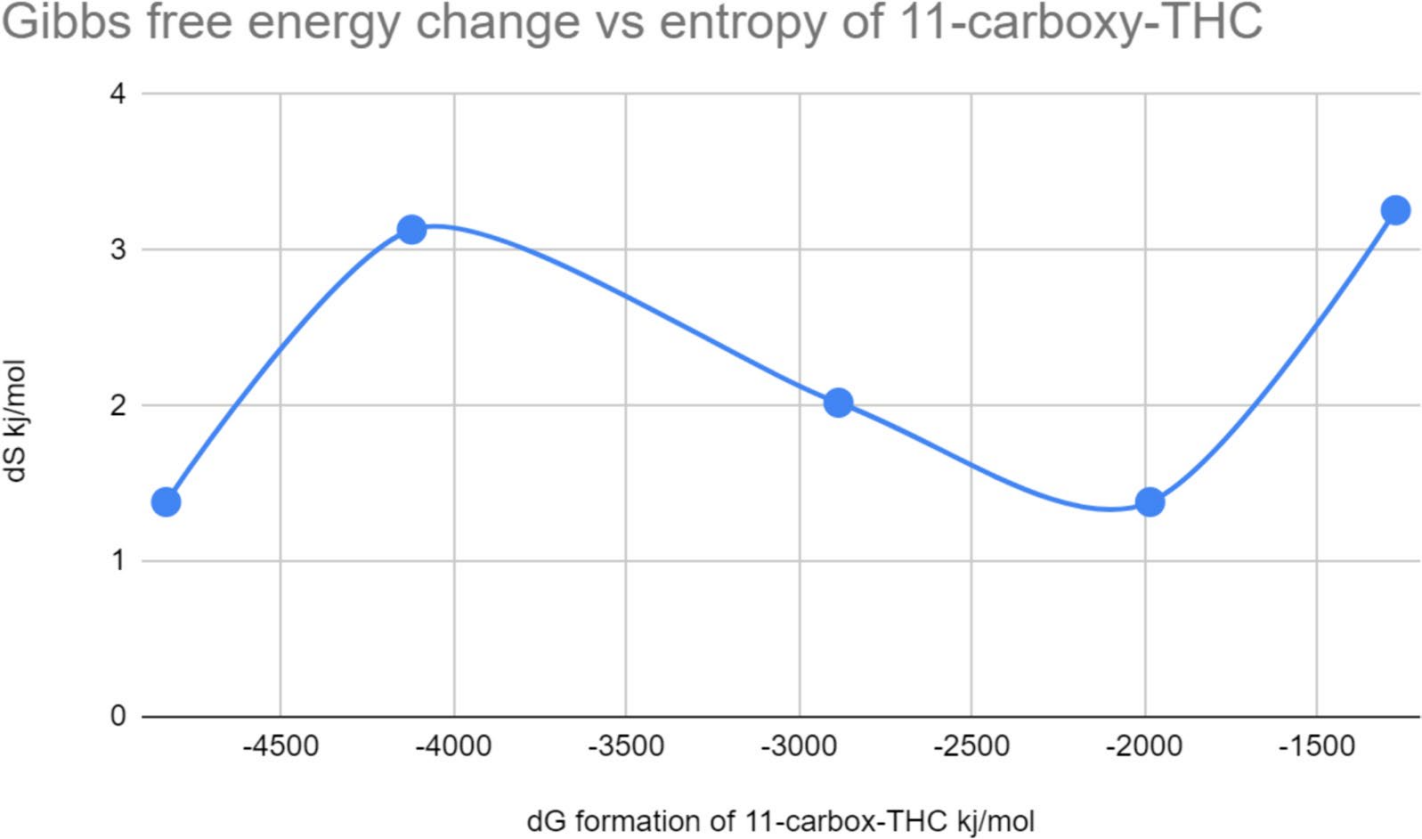
Free energy diagram of formation of 11-nor-carboxy-THC

**Fig. 8:**
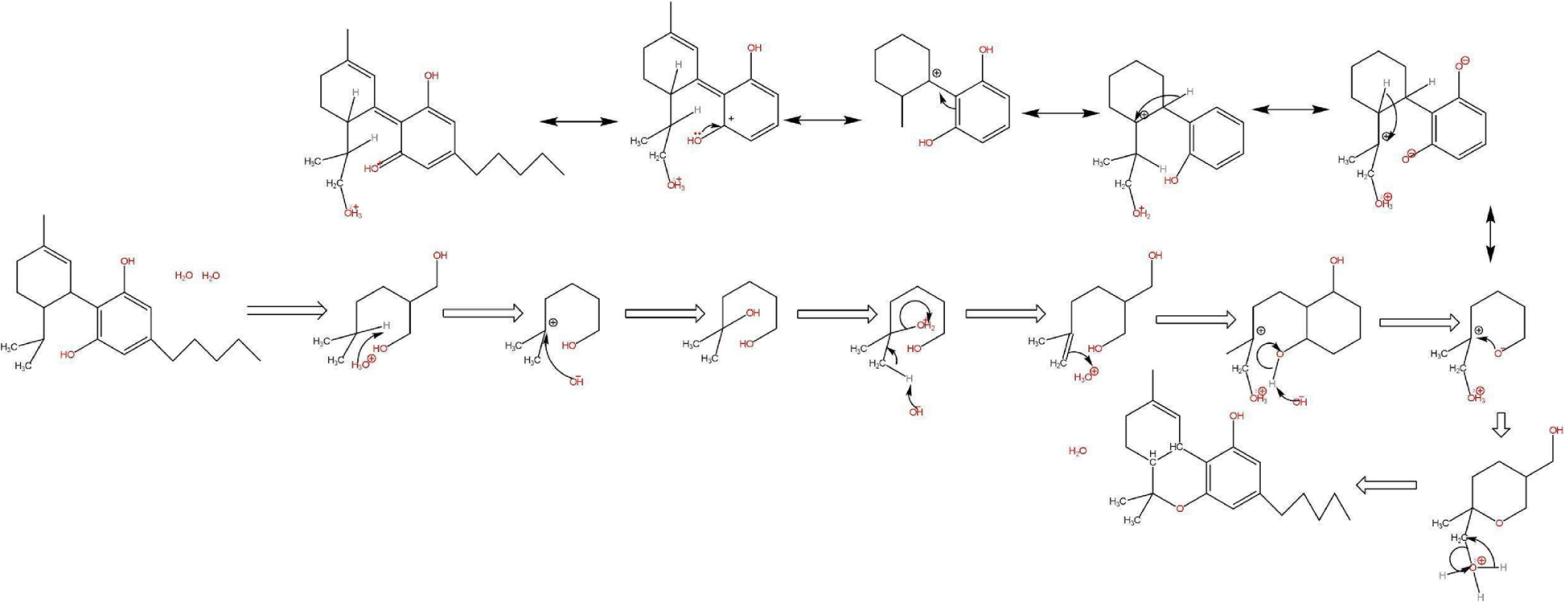
Formation of Δ^9^-THC from cannabidiol

Δ^9^ -THC to 11-OH THC:

$${{\Delta G}}_{{total}}={-6588.751\,{ kjmol}}^{-1}$$

$${{\Delta S}}_{{total}}= {+16.064\,{ kjmol}}^{-1}$$

Applying thermochemical equation: ΔG = -nFE.

As a 6 step electron transfer at equilibrium, ΔG_11-OH_ = -nFE_eq_

$${{E}}_{{eq}}= -({\Delta G})/{nF}= {-(-6588.749{kjmol}}^{-1}{/(6{mol \,x \,}96500{JC}-1{mol}}^{-1})) =+11.380{mV}$$

ΔG_redox_ = RT/nF -Ln K, where K = product/reactant each index was calculated as product/reactant in mechanism and of the cannabinoids considered with respect to the concentration of itself at equilibrium.

K = [11-OH-THC] /[Δ^9^-THC] [H_2_O] as equilibrium constant,

-RT Ln K = ΔG = ΔH -TΔS. Activated theory complex determines the rate constant from the thermodynamic functions of entropy and energy to determine the partition of energy within time that determines the chemical kinetic rate. This derives the rate constant of formation of products (Atkins 2001) (Tables 1, 2, 3 and 4).

**Table 1:** Thermodynamic energy changes of the mechanism Δ^9^-Tetrahydrocannabinol to 11-OH-THC

ΔH kjmol^−1^	ΔH sum kjmol^−1^	ΔG kjmol^−1^	ΔE mV	ΔS kjmol^−1^
-971	-971	-1272.407	13.186	3.257
-411.66	-1382.66	-711.191	7.370	1.381
-1960	-3342.66	-2259.531	23.415	6.574
-285.83	-3628.49	-584.939	6.062	0.959
-748.83	-4377.32	-1049.492	10.876	2.512
-411.66	-4788.98	-711.191	7.370	1.381

**Table 2:** Thermodynamic oxidation energy changes of the cannabinoids THC and CBD to CBN and CBND respectively

ΔH kjmol^−1^	ΔH sum kjmol^−1^	ΔG kjmol^−1^	ΔE mV	ΔS kjmol^−1^
-105	-105	-403.502	4.181	0.352
-933.83	-1038.83	-1235.112	12.799	3.132
-903	-1941.83	-1204.179	12.479	3.027
-411.66	-2353.49	-711.191	7.370	1.381
-411.66	-2765.15	-711.191	7.370	1.381
-317.66	-3082.81	-616.874	6.392	1.065

**Table 3:** Thermodynamic energy changes of the deactivation of Δ^9^-Tetrahydrocannabinol to 11-nor-carboxy-THC

ΔH kjmol^−1^	ΔH sum kjmol^−1^	ΔG kjmol^−1^	ΔE mV	ΔS kjmol^−1^
-971	-971	-1272.40675	13.186	3.256749958
-411.66	-1382.66	-711.1907144	7.370	1.380714406
-602	-1984.66	-902.1691179	9.349	2.019117894
-933.83	-2918.49	-1235.112081	12.799	3.132081167
-411.66	-3330.15	-711.1907144	7.370	1.380714406

**Table 4:** Thermodynamic energy changes of the reductive conversion of CBD to Δ^9^-Tetrahydrocannabinol

ΔH kjmol^−1^	ΔH sum kjmol^−1^	ΔG kjmol^−1^	ΔE mV	ΔS kjmol^−1^
-411	-411	-710.5285008	7.363	1.378500755
-411.33	-822.33	-710.8596076	7.366	1.37960758
-1485	-2307.33	-1788.130714	18.523	4.980714406
-182.85	-2490.18	-481.6132819	4.991	0.6132819051
-337.83	-2828.01	-637.1130874	6.602	1.133087372

Activation energy = -RT Ln K = ΔG = e.^−(G/RT)^

$${{E}}_{{A}}= {14.268{ \,kjmol}}^{-1}$$

$${{k}}_{{rate}}= {(k{T}/{h}){ e}}^{-({ dH }-{T dS}) /{RT}}$$

$${{k}}_{{rate}}= {(1.38066{JK}}^{-1} {{x }298.15{K}/ 6.626{x}10}^{-34}{Js}) {{x e}}^{-( -4788.98{ kj}/{mol }-(298.15{K x }16.064{ kj}/{mol}) /( 8.314{J}/{mol}/{K x }298.15{K})}$$

$${k}= {6.21255x10}^{35} {{x e}}^{1.93216} ={4.28854{x}10}^{36}{ seconds}$$

Where the rate expression for the chemical reaction can also be deduced from the mechanism where:

Δ^9^-THC to CBN:

$${{\Delta G}}_{{total}}= {-4882.049{ \,kjmol}}^{-1}$$

$${{\Delta S}}_{{total}}= {+10.338{ kjmol}}^{-1}{{K}}^{-1}$$

$${{E}}_{{A}}= {7.167{ kjmol}}^{-1}$$

$${{k}}_{{rate}}={4.4525{x}10}^{36}{ seconds}$$

As 6 step nucleophilic transfer to equilibrium:

$${{E}}_{{eq}}= -({\Delta G})/{nF}= {-(-6117.16{ \,kjmol}}^{-1}{(/6{mol \,x \,}96500{JC}}^{-1}{{mol}}^{-1})) = +10.565{mV}$$

$${{\Delta G}}_{{redox}}={RT}/{nF }-{Ln K},{ where \,K}={ product}/{reactant},$$

$${K}={[{CBN}]/[\Delta }^{9}-{THC}] [{{O}}_{2}] [{{H}}_{2}{O}]$$

As 5 step nucleophilic transfer to equilibrium:

$${{E}}_{{eq}}= -({\Delta G})/{nF}= {-(-4832.069{ \,kjmol}}^{-1}{(/5{mol \,x\, }96500{JC}}^{-1}{{mol}}^{-1})) = +10.015{mV}$$

K = product/reactant,

$${K}={[11-{nor}-{carboxy}-{THC}] /[\Delta }^{9}-{THC}] [{{O}}_{2}] [{{H}}_{2}{O}]$$

$${{\Delta G}}_{{total}}= {-4832.07{ \,kjmol}}^{-1}$$

$${{\Delta S}}_{{total}}= {+11.169{ \,kjmol}}^{-1}{{K}}^{-1}$$

$${{E}}_{{A}}={7.024{ \,kjmol}}^{-1}$$

$${{k}}_{{rate}}= {4.364{x}10}^{36}$$

$${{E}}_{{eq}}= -({\Delta G})/{nF}= {-(-4328.245{ \,kjmol}}^{-1}{(/5{mol \,x\, }96500{JC}}^{-1}{{mol}}^{-1})) = +8.970{mV}$$

$${{\Delta G}}_{{total}}={-4328.246{ \,kjmol}}^{-1}$$

$${{\Delta S}}_{{total}}={9.48519{ \,kjmol}}^{-1}{{K}}^{-1}$$

$${{E}}_{{A}}={5.73216{ \,kjmol}}^{-1}$$

$${{k}}_{{rate}}={3.561{x}10}^{36}{ seconds}$$

$$\text{K}= [{\Delta }^{9}-\text{THC}]/ [\text{CBD}] [{\text{H}}_{2}\text{O}]$$

## Discussion

The mechanism proposed intramolecular interactions with the alkene, the deduced synthesis yielded thermodynamic parameters that explains the probability of the difference in the cannabinoid reduction routes.

The restriction in the rotation of the cyclohexene ring of CBD was caused by the difference in the intramolecular OH- π bonds of the alkenes of both cyclic vs aliphatic interaction within the symmetry of the phenols in the aromatic ring (Daniels et al. 2023, Fig. 9). The redox routes of both cannabinoids were similar based on their functional groups however the location of the reaction was of importance which would determine the outcome of chemical reactions; spoilage through oxidation in storage to aromatization stability of CBN or CBND or reductions via conversion of THC to higher energies of CBN comparative to CBND, or metabolism in the animal body as reduction.

**Fig. 9:**
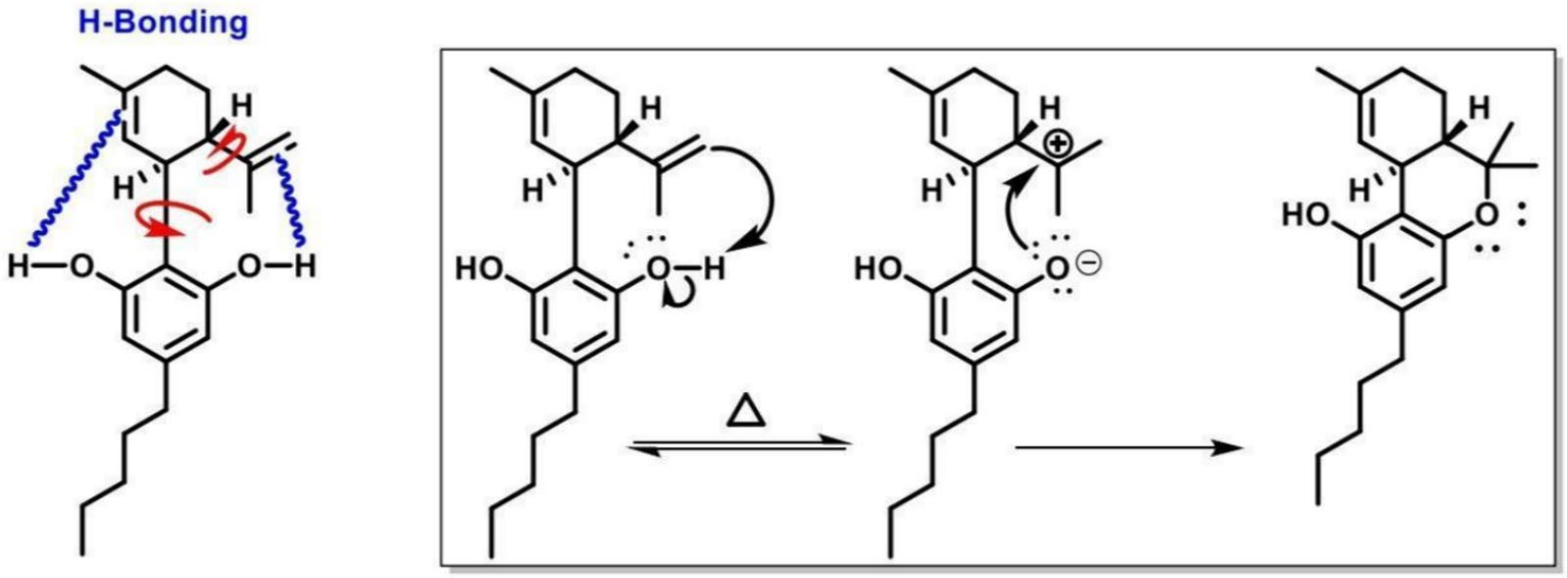
Proposed mechanism for the intermolecular isomerization of CBD to Δ^9^-THC

Where the reduction for CBD can occur as an intramolecular reaction converting to THC a different reduction can occur as metabolism in a mammalian body by the liver being more feasible and producing higher energies of thermodynamic parameters forming 7-OH-CBD to further metabolise to 7-COOH-CBD to deactivate the cannabinoid similar to THC of both isomers Δ^8^ and Δ^9^-THC. Oxidising the cannabinoids after reductive hydroxy intermediate formation through peroxide intermediates, enzymatic catalysis in the liver by the cytochrome P450 has the ability to further increase the rate routes of both cannabinoids metabolism to the carboxylic acids (Harvey and Brown 1991) from peroxides display a similar functional activities producing similar energetic parameters of pharmacokinetics.

**Fig. 10:**
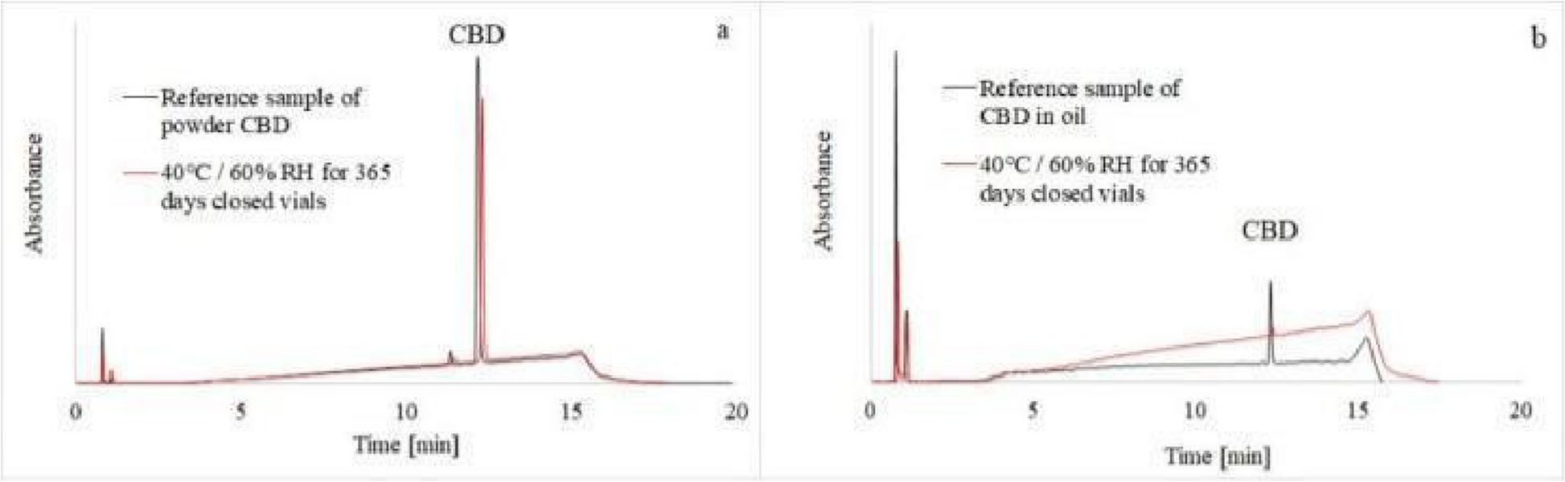
HPLC-UVchromatograms of CBD reference samples (black line) and CBD samples stored for 365 days (red line) in the stability chambers. The CBD powder was measured at 225 nm (a) and the CBD oil solution at 210 nm (b)

The problem of intramolecular conversion of CBD was observed in storage of CBD in sunflower oil resulting in detection of CBN in most samples however THC was not detected due to possible limitations. In addition, several oxidation products of CBD eluted at various retention times in partially degraded samples (Kosović et al. 2021). The oxidative degradation of both samples in oil and as powder is reflected in the functionality of CBN and of CBND having longer elution times being more aromatic with a stronger affinity with the non polar stationary phase. Where majority of the cannabinoids underwent protic conversion in sunflower oil via THC intermediate compared to relatively smaller concentrations and energies of CBND formation represented by Fig. 10. As CBD reduces to THC it is well understood that THC oxides to CBN as proposed by Turner and Elsohly.

**Fig. 11:**
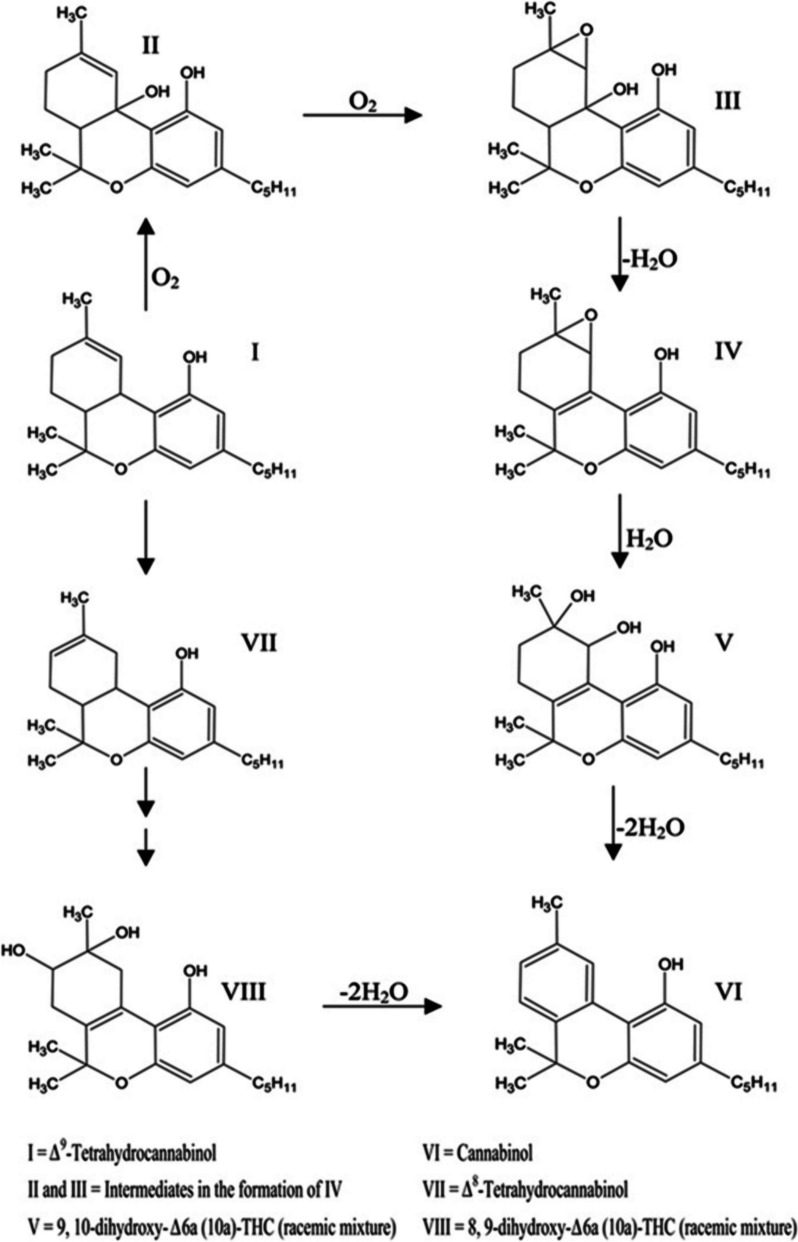
Proposed Pathway for Decomposition of THC (I) to Its Primary Degradant, Cannabinol (VI). (Adapted by Turner & ElSohly, 1979)

A pathway for the oxidation of Δ^9^-THC proposed by Turner and Elsohly using water and oxygen as reactants and product making CBN implies the cannabinoid as a lipid is undergoing oxidation with motifs of water in oxygen oxidation and proton reduction making water for free energy potential gains (Turner and Elsohly 1979). The mechanism in the proposal applied a quantized thermochemical mechanism to determine the electrochemical redox driven by the hydroxyl potential of water similarly as proposed by Turner (Fig. 11).

**Fig. 12:**
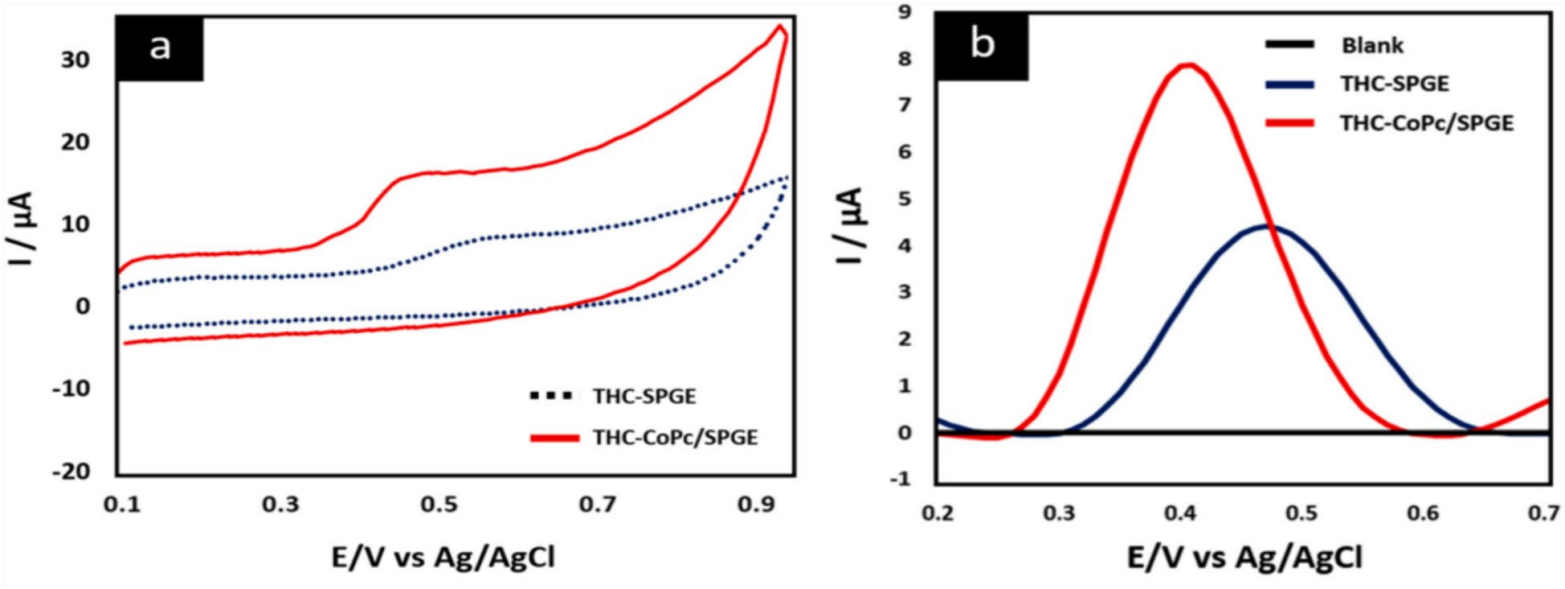
CVs and DPVs of 500 µg/mL THC in 0.1 M PBS buffer pH 7 at SPGE (blue line) and CoPc/SPGE (red line) on cPED. Buffer of 0.1 M PBS at pH 7 was measured using CoPc/SPGE (black line) as blank signal

**Fig. 13:**
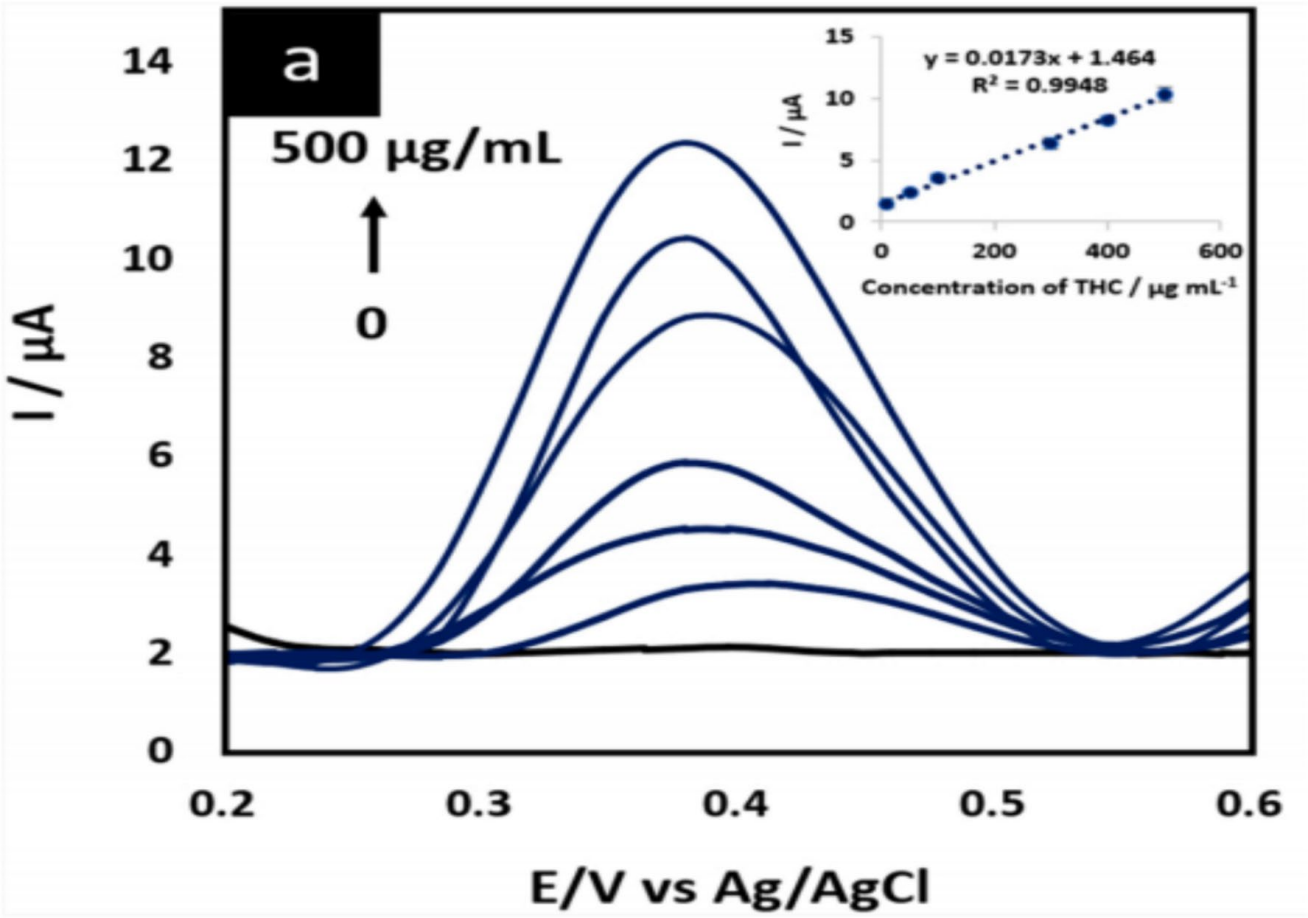
DPVs of THC (a) at a CoPc/SPGE in 0.1 M PBS pH 7 using cPED device at concentrations between 10 and 500 μg/mL. Inset: linear relationship of current signals vs. THC concentration. Experiment conditions: − 0.5 V vs. Ag/AgCl preconditioning potential, 15-s preconditioning time, 0.2 – 0.6 V vs. Ag/AgCl in the working range for THC determination

Pholsiri. T et al. had designed a chromatography paper-based electrochemical device as a means to electrochemically detect a chemical change through the use of a chemical sensor and actuator, to detect the chemical oxidation of THC. Where experimental values were detected by Pholsiri. T et al. in a buffer of pH 7 at equilibrium and a value of + 18 mV was detected in a 2 s phase pulse for THC in a chemical cell (Pholsiri, et al. 2022) represented in Figs. 12 and 13 the theoretical quantised value was calculated as + 10.565 mV as the oxidation formation of CBN using standard bond enthalpy data from Elements of Physical Chemistry.

**Fig. 14:**
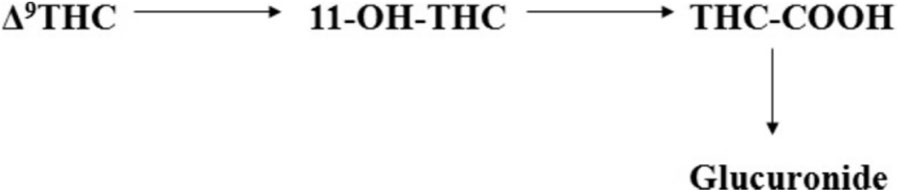
Metabolic route of -tetrahydrocannabinol (▵9_THC), its primary active metabolite 11-hydroxy-▵9-tetrahydrocannabinol (11-OH-THC) and the primary inactive metabolite, 11-nor-9-carboxy-▵ 9-tetrahydrocannabinol(THC-COOH)

The degradation activation energies of resin cannabinoids CBD and Δ^9^-THC were 3.86 kjmol^−1^ and 11.63 kjmol^−1^ respectively with degradation orders of zero and pseudo first orders respectively [ (Jaidee et al. 2021)]. From mechanistic extensive change activation energies were 5.73216 kjmol^−1^ and 7.167 kjmol^−1^ Δ^9^-THC from CBD and to CBN respectively, the parameter of intramolecular conversion were found to be within of each other as physical and theoretical values however CBN formation activation energies observed from Jaidee W. et al. were derived from increase in temperatures and different mass samples being smaller comparative to standard conditions which inturns influence the functions of energies as thermodynamic functions. It was observed that energies required for degradation were within the energies of formation suggesting the energies of oxidation were easily accessible to provide free energy releasing protons forming water. As a result of the freely feasible thermodynamic nature of conversion of CBD to Δ^9^-THC it was found that consumer products with active ingredients of CBD had contamination of Δ^9^-THC a psychotropic (Seccamani et al. 2021), the nature of C11 as an intermediate between 11-OH-THC, Δ^9^-THC and Δ^8^-THC isomerization proton catalysis in water driving stability of equilibriums the pH would be the determinant of the cannabinoids relative formations as chemical reduction. A proposed pathway of the deactivation of THC to nor-carboxy THC along with the final detectable metabolite (Sharma et al. 2012) (Fig. 14).

## Conclusion

Preservation of cannabinoids from oxidation can be synthesised by deactivation of the acidic proton of the phenol selectively with base as means of conversion to prevent the free energy change of spoilage and to prevent the conversion of non psychotropic CBD to Δ^9^-THC contamination. Metabolites from the consumption of cannabis follow the pharmacokinetic route both theoretically and from bioassays presented as clinical trials.

## Data Availability

Standard data had been provided by the elements of Physical Chemistry, Atkins 3rd edition in regards to bond energy data.

## Abbreviations

THC: Tetrahydrocannabinol
CBD: Cannabidiol
CBN: Cannabinol
CBND: Cannabinodiol
11-OH-THC: 11-Hydroxy tetrahydrocannabinol
11-nor-carboxy-THC: 11-Nor-carboxy tetrahydrocannabinol
COOH: Organic acid functional group
G: Gibbs free energy
S: Entropy
H: Enthalpy
mV: Millivolt
K: Equilibrium constant
n: Number of mols
F: Faraday constant
R: Gas constant
T: Temperature
k: Rate constant
*k*: Boltzman constant
h: Planck constant
e: Euler number
E: Energy
HPLC: High pressure liquid chromatography
UV: Ultraviolet
MS: Mass spectrometry
